# Percutaneous Retrieval of Left Atrial Appendage Closure Devices in Patients With Atrial Fibrillation: A Case Report

**DOI:** 10.3389/fcvm.2022.905344

**Published:** 2022-07-06

**Authors:** Saihua Wang, Juhua Zhang, Shuwen Hao, Luoning Zhu, Zhongping Ning, Zhihong Zhao

**Affiliations:** ^1^Department of Cardiology, Shanghai University of Medicine and Health Sciences Affiliated Zhoupu Hospital, Shanghai, China; ^2^Department of Social Medicine and Health Career Management, School of Public Administration, Fudan University, Shanghai, China

**Keywords:** atrial fibrillation, dislodgment, left atrial appendage closure, complications, retrieval

## Abstract

Left atrial appendage closure (LAAC) devices can be inadvertently released into unfavorable locations, which may allow them to migrate to a different position within the left atrial appendage or embolize from the heart into the aorta. In such instances, it can be challenging to remove the LAAC device. Here, we present two cases in which patients with atrial fibrillation experienced LAAC device exposure at an inappropriate site because of interventional operator error and device mismatch: (a) the LAAC device was dislodged into the aortic arch and retrieved percutaneously from the femoral artery route, and (b) in the left atrium, which was dislodged into the left atrium and retrieved *via* atrial transseptal puncture of the femoral vein.

## Introduction

Atrial fibrillation (AF) is one of the most common cardiac arrhythmias. Patients with AF are at high risk of ischemic stroke and require long-term or even life-long anticoagulation therapy. The left atrial appendage (LAA) is the site most prone to thrombosis in patients with AF. LAA occluders are not only an alternative method of anticoagulation therapy, but also a contraindication for oral anticoagulation, and several occluders, including those made in China, have been used in clinical practice (1). The device detachment after left atrial appendage closure (LAAC) has a low incidence rate but carries a fatal risk. Herein, we report two cases of LAAC device detachment in which the LAAC devices were removed *via* the percutaneous femoral artery or femoral vein retrieval. We also analyzed the possible treatment strategies following a review of the available literature.

## Case Description

**FIGURE 1:**
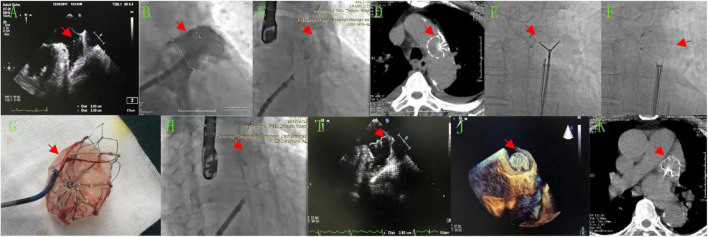
After LAAC, the occluder fell off into the aortic arch and was feathered out, before the 33-mm Watchman device was occluded again. **(A,I,J)** LA TEE images. **(A)** Preoperative LAA: ostial of 25–30 mm and an effective depth of 28 mm. **(I,J)** Two-dimensional and 3-dimensional images after the second closure with the Watchman device. **(B,C,E,F,H)** DSA images. **(B)** Preoperative LAA with an opening of 28 mm and a depth of 31 mm. **(C)** After occlusion with the 33-mm Watchman device. **(E,F)** Endoscopic Raptor forceps were used to clamp the umbrella trabeculae and drag it into the sheath. **(H)** Second closure with the Watchman device. **(D,K)** Chest CT image. **(D)** Occluder in the aortic arch. **(K)** LAAC with the Watchman device. **(G)** Modified occluder.

**Case 1** involved a 69-year-old male patient with a history of persistent AF, hypertension, diabetes, cerebral embolism, and transient ischemic attack for > 10 years. The patient had a history of taking warfarin but had self-discontinued the medication. Transthoracic echocardiography revealed the following findings: left atrium (LA), 44 mm; left ventricle (LV) end-systole, 46 mm; LV end-diastolic, 55 mm; LV ejection fraction, 58%; mitral calcification with minimal regurgitation; and minimal aortic regurgitation. Both the CHA_2_DS_2_-VASc and HAS-BLED scores were 6 points. LA computed tomography angiography (CTA) excluded LA thrombus. The patient underwent a combination of catheter ablation and LAAC in a single procedure. Transesophageal echocardiography (TEE) measured an LAA ostial of 25–30 mm and an effective depth of 28 mm (Figure 1A). Digital subtraction angiography (DSA) measured the LAA opening as 28 mm and the depth as 31 mm (Figure 1B). The Watchman 33-mm device (Boston Scientific, Marlborough, MA, United States) was released, with a compression ratio of 15–24% and no residual peri-device leak (Figure 1C), then proceeded to complete AF pulmonary vein isolation ablation. After returning to the ward, the patient developed a cough and shortness of breath. Bedside echocardiography did not show the LAA occluder, and a chest CT scan revealed that the occluder was in the aortic arch (Figure 1D). A 5-French (Fr) Tig angiography catheter (Terumo Corporation, Tokyo, Japan) was sent through the radial artery to the aortic arch for relative fixation of the occluder. Next, the 5-Fr pigtail angiography catheter was sent through the 6-Fr sheath of the left femoral artery to the right femoral artery puncture site to delineate to ensure that the puncture site was located in the middle of the femoral artery. After a successful puncture of the right femoral artery, two ProGlide vascular staplers (Abbott, Chicago, IL, United States) were pre-set at the puncture site. A Watchman guide system 16-Fr sheath (Boston Scientific) was sent under the occluder through the femoral artery, and a 7-Fr guide catheter was sent through the sheath to the occluding umbrella. Next, the Amplatzer gooseneck snare, a 20 or 40-mm mesh basket guidewire (Medtronic, Minneapolis, MN, United States), was caught and covered. The occluder was pulled down to the descending aorta. Then, the 2.4-mm × 20-cm Raptor grasping device (Raptor US Endoscopy, Mentor, OH, United States) was advanced through the 16-Fr sheath into the LA to clamp the trabeculae of the umbrella. After intrathecal injection of ice-cold 0.9% saline, the vascular sheath was pushed, the occluding umbrella was recovered into the sheath (Figures 1E,F), and the Watchman occluder was removed (Figure 1G). When the vascular sheath was withdrawn, the femoral artery was sutured with ProGlide at the puncture site of the femoral artery. Then, the patient was returned to the ward safely, and LAAC was performed again 5 months later. In this later surgery, the 33-mm Watchman was placed, and DSA and TEE confirmed that the Watchman device was properly positioned (Figures 1H–J). The compression ratio was 15–20%, and there was no residual leakage. The postoperative follow-up re-examination of TEE was normal. Six months later, a chest CT showed that the LAA occluder was in a good position (Figure 1K).

**FIGURE 2:**
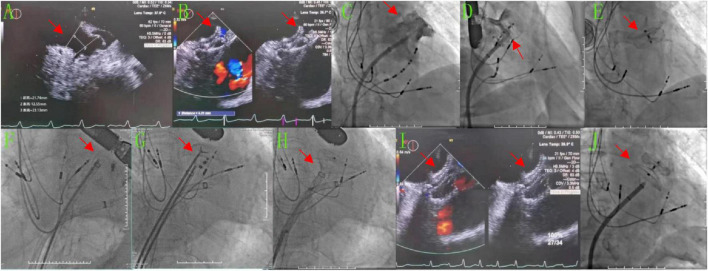
After LAAC, the occluder detached into the LA and was retrieved, before the Lacbes 2834 device was occluded again. **(A,B,I)** TEE images. **(A)** Preoperatively, the diameter of the LAA opening was 18.3–22 mm and the diameter of the anchoring area was 16.5–24.4 mm. **(B)** The Lacbes 2834 was placed in LAA, the fixed column was exposed, and the residual shunt at the upper edge was 4.3 mm. **(I)** After the second LAAC procedure with Lacbes 2636, the covering disc adhered well and had no residual shunt. **(C–H,J)** DSA images. **(C)** Preoperatively, the diameter of the LAA change opening was 28.56 mm and the diameter of the anchoring area was approximately 31.47 mm. **(D)** After closure with the Lacbes 2834 device. **(E)** The Lacbes fell into the LA. **(F–H)** Endoscopic Raptor forceps were used to clamp the umbrella trabeculae and drag them into the sheath. **(J)** After the second LAAC procedure with the Lacbes 2636 device.

**Case 2** involved an 86-year-old male patient with a history of persistent AF for 3 years and an implanted pacemaker because of bradyarrhythmia for 6 years. His CHA_2_DS_2_-VASc score was 5 points, and his HAS-BLED score was 2 points. After cryoablation, LAAC was performed, in which the diameter of the LAA opening as measured by TEE was 18.3–22 mm and the anchoring zone was 16.5–24.4 mm (Figure 2A). The LAA showed a chicken wing shape. The diameter of the opening of the LAA as measured by DSA was 28.6 mm, and the anchoring area was 31.5 mm (Figure 2C). A Lacbes 2834 device was delivered (Figure 2D). TEE showed that the shoulder of the fixed column was mildly exposed, and the residual shunt at the upper edge was 4.3 mm, which was within the allowable range. The occluder was slowly released after pulling it steadily (Figure 2B). After returning to the ward, the patient had no chest tightness or shortness of breath, but he did complain of drowsiness, and his blood pressure dropped to 58/41 mmHg, with a heart rate of 60 beats/min. Subsequently, dopamine 20 mg was administered intravenously while increasing the rate of fluid replacement to maintain blood pressure. Bedside echocardiography showed that the occluder had drifted in the LA, and the possibility of the occluder detaching was considered. Emergency LAA occlusion umbrella capture was performed under general anesthesia with endotracheal intubation and ventilator-assisted breathing. Under DSA, the occluder was seen floating in the LA (Figure 2E). The atrial septal puncture was performed twice through the right femoral vein route. Two 14-Fr cryoablation steerable sheaths were sent to the LA. A pigtail catheter (Terumo) was delivered across the mitral valve to prevent the occluder from crossing the mitral valve and traveling into the LV. The Raptor grasping device and pigtail catheters were sent through another sheath, and the latter was used to attempt to fix the occluder in an appropriate position, i.e., coaxial alignment of the jaws of the Raptor grasping device to the center of the Lacbes disc. Once the disc was grasped, sustained traction initially dislodged the proximal disc from the LA, and the occluder was pulled back to the sheath (Figures 2F–H); then, the Lacbes 2636 device was placed in the LAA (Figures 2I,J). After discharge, warfarin was used to control the international normalized ratio (INR) to 2–3. Results of the 3-month postoperative follow-up re-examination of TEE were normal. Long-term oral administration of clopidogrel antiplatelet drugs was enacted. During the follow-up period, the patient’s quality of life was good, and persistent follow-up will be conducted.

## Discussion

The LAA is the most common site of atrial thrombus in AF (2). LAAC is a viable strategy for stroke risk reduction in patients with non-valvular AF who can tolerate systemic oral anticoagulation therapy but are unsuitable candidates for long-term anticoagulation (3). Occluder detachment is one of the most serious complications of LAAC. Complications have been reported in the ASAP study, which included thoracotomy and percutaneous interventional methods (4, 5). Most occluder detachments occur during the perioperative period but can also happen any time from hospital discharge to 1 year later (6, 7). LAA detachment is usually asymptomatic unless the patient is hemodynamically affected; for example, in our case 2, the occluder fell into the LA and hung on the mitral valve, resulting in a drop in blood pressure. If the occluder lodges in the mitral valve or aortic valve, it may lead to sudden death.

We described two cases of percutaneous retrieval of dislodged LAAC devices from the LA and aorta through the femoral artery and femoral vein routes, respectively. In case 1, after LAAC, the ablation catheter was touched and the occluder fell out of the LAA during pulmonary vein isolation. In case 2, after cryoablation, LAAC was performed, and because the selected occluder size is small for the LAA, it fell out of the LAA. The Raptor grasping device was designed by gastroenterologists for retrieving foreign bodies, which has a hybrid jaw configuration that combines alligator and rat tooth capabilities into a single device, increasing its gripping ability. Its design played a key role in tightly grabbing and withdrawing the devices in our cases. However, caution is recommended while engaging the LAAC device due to the risk of vascular wall damage from inadvertently grasping the vascular wall; in this series, 14-Fr and 16-Fr sheaths provided a large enough lumen and support to accommodate full device retrieval.

The findings of the concrete analysis of 18 cases of LAA occluder detachment published from 2013 to 2021 (4, 6–17) are as follows: the duration of occluder detachment occurs from the day the patient is discharged to 1 year later; among these patients, 70% are asymptomatic, while 30% have hemodynamic instability, arrhythmia, and cerebral infarction. Reasons for occluder detachment include the variable shape of the LAA, the fixed type of occluder, the unsuitability of the occluder for the LAA, and no clear reason. The positions where the occluder falls off include the aorta (32%), the LV (29%), and the LA (25%), and looseness in the LAA also contributes to occluder detachment (14%), which can cause mitral valve damage, hemodynamic instability, or ventricular arrhythmia. The following procedures can be performed simultaneously: thoracotomy to remove the LAA occluder, aortic valve replacement, mitral valve replacement or repair, atrial fibrillation (AF) Maze surgery or ablation, and LAA resection or suture or clipping. The occluder can be percutaneously retrieved from the LA cavity, LAA, or the LV cavity, and even from the aorta. When percutaneous retrieval is expected to be difficult, surgical retrieval should be actively considered (3).

The materials used for percutaneous retrieval of occluders include a 14-Fr cryoablation Agilis sheath, 16-Fr LAA occluder introducer sheath, and 4-Fr Agilis sheath for mitral valve clipping (MitraClip; Abbott), or 27-Fr sheath used for wireless pacemaker (Micra; Medtronic). The capture devices include the gooseneck snare, Ensnare (Merit Medical Inc., South Jordan, UT, United States), a self-made capture device, or Raptor grasping device. It should be noted that the iatrogenic patent foramen ovale was caused by percutaneous retrieval of the occluder through the fossa ovalis.

In conclusion, we herein reported two case studies of LAA occluder detachment where the occluder dislodged and drifted in the aortic arch in one case and the LA in the other. Both occluders were successfully retrieved *via* a femoral artery or femoral vein route, respectively. The causes, characteristics, and removal methods of the LAA occluder were summarized by combining our cases with those reported previously.

## Data Availability Statement

The original contributions presented in this study are included in the article/Supplementary Material, further inquiries can be directed to the corresponding author/s.

## Ethics Statement

The studies involving human participants were reviewed and approved by the China Medical University. The patients/participants provided their written informed consent to participate in this study. Written informed consent was obtained from the individual(s) for the publication of any potentially identifiable images or data included in this article.

## Author Contributions

ZZ participated in research design and data acquisition and analysis. SH, SW, JZ, and ZZ participated in the writing of the manuscript. JZ, LZ, and ZN participated in the performance of the research. All authors have read and approved the manuscript.

## Conflict of Interest

The authors declare that the research was conducted in the absence of any commercial or financial relationships that could be construed as a potential conflict of interest.

## Publisher’s Note

All claims expressed in this article are solely those of the authors and do not necessarily represent those of their affiliated organizations, or those of the publisher, the editors and the reviewers. Any product that may be evaluated in this article, or claim that may be made by its manufacturer, is not guaranteed or endorsed by the publisher.

## References

[1] He B Jiang LS. [A brief discussion: the impact of “2019 Chinese society of cardiology (CSC) expert consensus statement on left atrial appendage closure in the prevention of stroke in patients with atrial fibrillation” on the evolution of technical development of LAAC in China]. *Zhonghua Xin Xue Guan Bing Za Zhi.* (2021) 49:212–6. 10.3760/cma.j.cn112148-20210131-00113 33706453

[2] Schotten U Verheule S Kirchhof P Goette A. Pathophysiological mechanisms of atrial fibrillation: a translational appraisal. *Physiol Rev.* (2011) 91:265–325. 10.1152/physrev.00031.2009 21248168

[3] Chinese Society of Cardiology of Chinese Medical Association, Editorial Board of Chinese Journal of Cardiology. [2019 Chinese society of cardiology (CSC) expert consensus statement on left atrial appendage closure in the prevention of stroke in patients with atrial fibrillation]. *Zhonghua Xin Xue Guan Bing Za Zhi.* (2019) 47:937–55. 10.3760/cma.j.issn.0253-3758.2019.12.002 31877589

[4] Gupta P Szczeklik M Selvaraj A Lall KS. Emergency surgical retrieval of a migrated left atrial appendage occlusion device. *J Card Surg.* (2013) 28:26–8. 10.1111/jocs.12038 23211043

[5] Reddy VY Mobius-Winkler S Miller MA Neuzil P Schuler G Wiebe J Left atrial appendage closure with the Watchman device in patients with a contraindication for oral anticoagulation: the ASAP study (ASA plavix feasibility study with Watchman left atrial appendage closure technology). *J Am Coll Cardiol.* (2013) 61:2551–6. 10.1016/j.jacc.2013.03.035 23583249

[6] Turagam MK Neuzil P Dukkipati SR Petru J Skalsky I Weiner MM Percutaneous retrieval of left atrial appendage closure devices with an endoscopic grasping tool. *JACC Clin Electrophysiol.* (2020) 6:404–13. 10.1016/j.jacep.2019.11.015 32327074

[7] Martinez-Lopez D de Villarreal Soto JE Mosquera VMO Gil AF. Emergency surgical retrieval of a migrated LAmbre device through the mitral valve. *Eur J Cardiothorac Surg.* (2021) 60:1475–6. 10.1093/ejcts/ezab342 34331063

[8] Nunes A Pissarra D Tavares Silva M Almeida PB Silva JC Maciel MJ. Embolization of a left atrial appendage closure device. *Rev Port Cardiol (Engl Ed).* (2021) 40:247–8. 10.1016/j.repc.2019.12.010 33483176

[9] Maan A Turagam MK Dukkipati SR Reddy VY. Percutaneous extraction of a migrated WATCHMAN device after seven months. *J Innov Card Rhythm Manag.* (2021) 12:4572–4. 10.19102/icrm.2021.120701 34277127 PMC8221633

[10] Lubis AC Iqbal M Munawar DA Hartono B Munawar MA. Simple percutaneous retrieval technique for an embolized Watchman left atrial appendage closure device in the thoracic aorta using a homemade snare. *Int Heart J.* (2021) 62:1153–5. 10.1536/ihj.20-790 34544965

[11] Tschishow WN Israel CW. [Dislodgement of a left atrial appendage occluder : step-by-step management by retrograde extraction with a “home-made snare” and two sheaths]. *Herzschrittmacherther Elektrophysiol.* (2020) 31:430–3. 10.1007/s00399-020-00726-3 33034760

[12] Sun X Hong D Liu H Li H. Acute mitral valve injury following percutaneous left atrial appendage occlusion: a case report and literature review. *Heart Surg Forum.* (2020) 23:E743–5. 10.1532/hsf.3157 33234217

[13] Takayuki G Grimmig O Soren J Dirk F. Asymptomatic dislocation of a Watchman left atrial appendage occluder. *Asian Cardiovasc Thorac Ann.* (2019) 27:394–5. 10.1177/0218492318805620 30282462

[14] El-Gabry M Shehada SE Wendt D Mourad F. Emergent surgical removal of a migrated left atrial appendage occluder. *Eur J Cardiothorac Surg.* (2018) 54:191–2. 10.1093/ejcts/ezy005 29385439

[15] Sanhoury M Fassini G Dello Russo A Lumia G Bartorelli A. Early dislodgment and migration of a left atrial appendage closure device. *Am J Cardiol.* (2017) 120:1905–7. 10.1016/j.amjcard.2017.07.077 28917494

[16] Lee OH Lee H Kim JS. Successful retrieval of a dislodged left atrial appendage closure device. *JACC Cardiovasc Interv.* (2017) 10:98–100. 10.1016/j.jcin.2016.10.044 28057291

[17] Pisani P Sandrelli L Fabbrocini M Tesler UF Medici D. Left-atrial-appendage occluder migrates in an asymptomatic patient. *Tex Heart Inst J.* (2014) 41:443–4. 10.14503/THIJ-13-3173 25120404 PMC4120514

