# Supplementary figures and images for: Percutaneous Retrieval of Left Atrial Appendage Closure Devices in Patients With Atrial Fibrillation: A Case Report
Source: Front Cardiovasc Med. 2022 Jul 6;9:905344. doi: 10.3389/fcvm.2022.905344 (PMC9301371; doi:10.3389/fcvm.2022.905344)

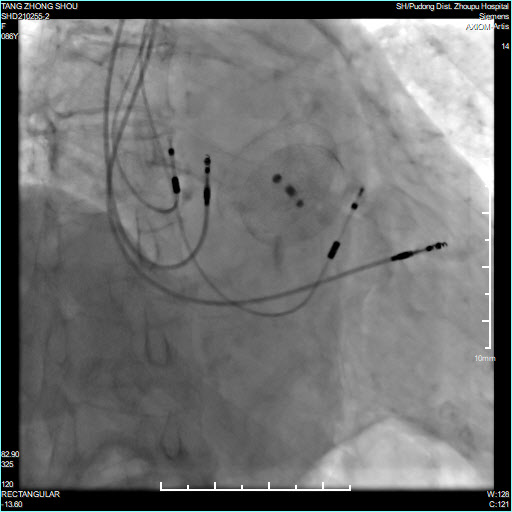

Supplement: Supplementary file 1 [file Image_1.jpeg]
